# Anisotropic Diffusion in Driven Convection Arrays

**DOI:** 10.3390/e23030343

**Published:** 2021-03-14

**Authors:** Yunyun Li, Vyacheslav R. Misko, Fabio Marchesoni, Pulak K. Ghosh

**Affiliations:** 1 Center for Phononics and Thermal Energy Science, Shanghai Key Laboratory of Special Artificial Microstructure Materials and Technology, School of Physics Science and Engineering, Tongji University, Shanghai 200092, China; yunyunli@tongji.edu.cn (Y.L.); veaceslav.misco@vub.be (V.R.M.); 2 μFlow Group, Department of Chemical Engineering, Vrije Universiteit Brussel, 1050 Brussels, Belgium; 3 Dipartimento di Fisica, Università di Camerino, I-62032 Camerino, Italy; 4 Department of Chemistry, Presidency University, Kolkata 700073, India; pulak.chem@presiuniv.ac.in

**Keywords:** brownian motion, classical transport, convection rolls, advection enhanced diffusion

## Abstract

We numerically investigate the transport of a Brownian colloidal particle in a square array of planar counter-rotating convection rolls at high Péclet numbers. We show that an external force produces huge excess peaks of the particle’s diffusion constant with a height that depends on the force orientation and intensity. In sharp contrast, the particle’s mobility is isotropic and force independent. We relate such a nonlinear response of the system to the advection properties of the laminar flow in the suspension fluid.

## 1. Introduction

Under quite general conditions, the fluctuation-dissipation theorem relates the response of a system to an external perturbation with its equilibrium dynamics [1]. However, the theorem’s predictions do not apply either in the nonlinear response regime, when higher order corrections to the response functions grow appreciably, or out of equilibrium, when the detailed balance is broken and currents flow across the system [2]. The theorem can then be generalized in various ways [3].

An archetypal example of nonlinear response is represented by the dynamics of a tracer particle moving in a complex medium under the action of an external force $\vec{F}$. Two common response quantifiers are the tracer’s mobility, μ, and its diffusion constant, *D*, in the force direction. Upon increasing *F*, both observables are strongly affected by the interaction between the tracer and its surrounding medium. This approach has inspired active microrheology techniques [4], whereby the local and bulk mechanical properties of a complex fluid, such as emulsions, suspensions, polymers, and micellar solutions, are extracted from the motion of probe particles embedded within it.

Among the most surprising effects of the nonlinear response in nonequilibrium systems are negative differential mobility [5] and absolute negative mobility [6]. The former is due to the changes of the medium caused by the driven tracer, whereas the latter results from a more subtle combination of time memory, spatial asymmetry, and driving fields [7]. Negative differential mobility is a relatively more frequent phenomenon [8]. It has been detected in geometries characterized by the emergence of entropic channels, with a driven particle squeezing its way through a periodic array of rigid pores. These are effective barriers of an entropic nature [9], which oppose the action of the external drive with height dependent on the drive itself [10]. Another instance of negative differential mobility has been reported for driven tracers in crowded environments [11,12], whereas the magnitude and extension of the effect is controlled by the density and diffusion time of the obstacles.

A somehow related problem is particle transport in arrays of convection rolls. Here, geometric constraints [13,14,15] are replaced by advection cells. This is a recurrent problem in today’s nanotechnology [16,17] with promising applications in chemical engineering [18]. Under quite general conditions, the combined action of advection and thermal fluctuations in the suspension fluid is known to accelerate particle diffusion, an effect known as advection enhanced diffusion (AED) [19,20]. However, the effects of an external drive on the mobility and diffusion constant of an advected passive tracer have been scarcely investigated in the current literature [21].

**Figure 1:**
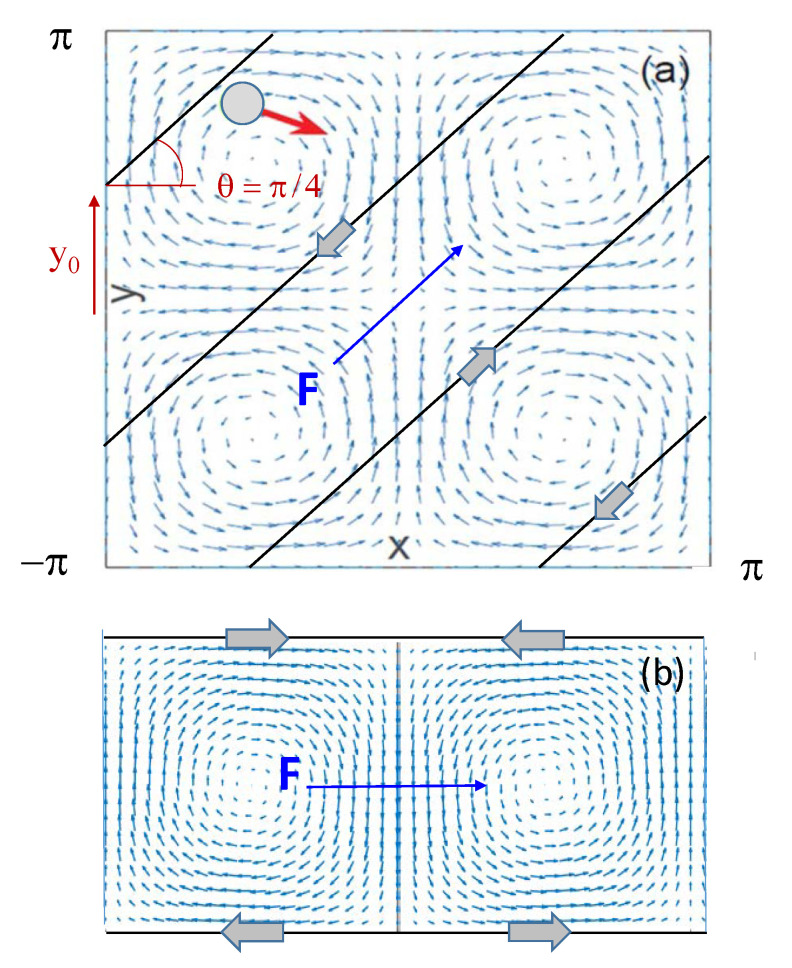
Driven particle in a square convection array of Equation (1) with L=2π. The force $\vec{F}$ (dark blue arrow) is oriented: (**a**) diagonal to the convection rolls (θ=π/4); (**b**) parallel to the *x* axis (θ=0). Parallel periodic channels are delimited by solid black lines; arrows denote the advection velocity field, $\vec{u}$ (light-blue), and the drag orientation along the channel edges (thick, gray).

Let us consider a massless Brownian particle suspended in a square lattice of counter-rotating convection rolls with periodic stream function (Figure 1),

(1) $$\psi \left(x,y\right)=\left(U_{0}L/2\pi \right)\sin\left(2\pi x/L\right)\sin\left(2\pi y/L\right).$$

Here, *L* is the size of the flow unit cell, $U_{0}$ the maximum advection speed at the roll separatrices, $\Omega_{L}=2\pi U_{0}/L$ the maximum vorticity at their centers, and $D_{L}=U_{0}L/2\pi$ an intrinsic flow diffusion constant. The mobility of an overdamped Brownian particle in an incompressible flow is μ=1, regardless of the applied force $\vec{F}$ [21]. Conversely, our numerical simulations show that the diffusion constant depends on both the modulus and the orientation of $\vec{F}$. The nonlinear nature of the system response is apparent since μ is isotropic in the plane *x*-*y*, whereas *D* is strongly anisotropic, with maxima in the diagonal direction. Moreover, *D* exhibits huge peaks for *F* of the order of the advection drag, $U_{0}$, which we interpret as excess diffusion peaks due to the interplay of drive and advection along the roll separatrices of ψ(x,y).

This paper is organized as follows. In Section 2, we present our model and briefly recap recent results on AED in unbiased laminar flows [22]. In Section 3, we summarize some new numerical results obtained in the presence of an external planar force of tunable intensity and orientation. We focus on the profile of the D(F) curves, namely on their horizontal asymptotes and remarkable excess diffusion peaks. Three drive orientations deserve special attention, namely diagonal with respect to the main axes of the square roll array and parallel to them. Such special cases are discussed in Section 4 and Section 5, respectively. In Section 6, we anticipate future venues for this line of research.

## 2. Model

A point-like Brownian particle diffusing in the flow with stream function ψ(x,y), Equation (1), obeys the Langevin equations,

(2) $$\begin{array}{c} \dot{x}=u_{x}+F\cos\theta +\xi_{x}\left(t\right),\;\;\;\;\dot{y}=u_{y}+F\sin\theta +\xi_{y}\left(t\right), \end{array}$$

where *x* and *y* are the particle’s coordinates, $\vec{u}=\left(u_{x},u_{y}\right)=\left(\partial_{y},-\partial_{x}\right)\psi$ is the incompressible advection velocity vector, $\vec{\nabla }\cdot \vec{u}=0$, and $\vec{F}=F\left(\cos\theta ,\sin\theta \right)$ a tunable, uniform field, termed here “force”, oriented at an angle θ with respect to the *x* axis. As illustrated in Figure 1a, the array unit cell consists of four counter-rotating convection rolls. The random sources, $\xi_{i}\left(t\right)$ with i=x,y, are stationary, independent, delta-correlated Gaussian noises, $\langle \xi_{i}\left(t\right)\xi_{j}\left(0\right)\rangle =2D_{0}\delta_{ij}\delta \left(t\right)$, modeling equilibrium thermal fluctuations in a homogeneous, isotropic medium. In our notation, $D_{0}$ coincides with the particle’s free diffusion constant in the absence of advection. Upon adopting the flow parameters, *L* and $\Omega_{L}^{-1}$, as convenient length and time units, the only remaining tunable parameters are the noise strength, $D_{0}$ (in units of $D_{L}$), and the force parameters, *F* (in units of $U_{0}$) and θ.

The stochastic differential Equation (2) were numerically integrated by means of a standard Mil’shtein scheme [23]. To ensure numerical stability, numerical integration was performed using a very short time step, $10^{-5}$–$10^{-4}$. The stochastic averages reported here were computed over at least $10^{7}$ samples (trajectories). Computing the asymptotic diffusion constants requires extra caution, because at low noise, the advected particle may take an exceedingly long time to exit a convection roll [24]. Indeed, since the external force $\vec{F}$ breaches the $C_{4}$ symmetry of ψ(x,y), we compute the diffusion constants along the *x* and *y* axes,

(3) $$D_{x}=\lim_{t\to \infty }\langle {\left[\Delta x\left(t\right)\right]}^{2}\rangle /2t,\;\;\;D_{y}=\lim_{t\to \infty }\langle {\left[\Delta y\left(t\right)\right]}^{2}\rangle /2t,$$

where Δx(t)=x(t)−〈x(t)〉, Δy(t)=y(t)−〈y(t)〉, and 〈…〉 denotes a stochastic average. These two diffusion constants will be investigated as distinct functions of *F* and θ in lieu of the diffusion constant in the force direction, D(F), introduced in Section 1. Of course, in view of the $C_{4}$ symmetry of the stream function of Equation (1), one proves immediately that $D_{x}\left(\theta \right)=D_{x}\left(-\theta \right)$ and, more importantly, $D_{y}\left(F,\theta \right)=D_{x}\left(F,\pi /2-\theta \right)$.

Subject to thermal fluctuations of strength $D_{0}$, an unbiased advected particle with F=0 undergoes normal diffusion with an asymptotic diffusion constant, $D=D_{x}=D_{y}$. AED takes place at high Péclet numbers, $\mathrm{Pe}=D_{L}/D_{0}\gg 1$, whereby *D* turns out to be larger than the free diffusion constant, $D_{0}$. This effect has been explained [19,20,21,25,26,27] by noticing that, at low noise, an unbiased particle jumps between convection rolls thanks to advection, which drags it along the outer layers of the convection rolls, or flow boundary layers (FBLs), centered around the ψ(x,y) separatrices. Thermal diffusion across such narrow FBLs favors particle roll jumping, thus enhancing spatial diffusion [24].

At zero bias, the asymptotic diffusion constant, *D*, changes from $D=\kappa \sqrt{D_{L}D_{0}}$ for Pe≫1 to $D=D_{0}$ for Pe≪1. The constant κ depends on the array geometry and boundary conditions [19,26]. For the unbiased, square free-boundary convection array of Equation (1), κ≃1.07 [19], consistent with previous numerical results [24]. The crossover between these diffusion regimes is well localized around $D_{0}≃D_{L}$ [27]; hence, AED occurs for $D_{0}<D_{L}$. Many numerical and experimental papers support the FBL-based interpretation of AED [28,29,30,31,32].

The relevant mobility functions are computed by taking the limits:

(4) $$\mu_{x}=\lim_{t\to \infty }\langle x\left(t\right)\rangle /Ft,\;\;\;\mu_{y}=\lim_{t\to \infty }\langle y\left(t\right)\rangle /Ft.$$

We numerically checked that $\mu_{x}=\mu_{y}=1$ for any choice of *F* and θ, as to be expected in the notation of Equation (2).

In a recent paper, we investigated the effect of a longitudinal force, say $F=F_{x}$, on the trajectories of a tracer particle [22]. For small *F* values, the tracer is advection dragged along the boundaries of a convection roll until, after completing several rounds, it jumps into an adjacent one (preferably in the force direction). The FBL width, δ, is of the order of the length diffused by a free Brownian particle during a full convection round, that is $\delta ={\left(D_{0}/\Omega_{L}\right)}^{1/2}$ [19,20]. Upon increasing *F*, the tracer’s circulation along the roll boundaries stops, while its trapping inside the convection rolls grows but is short lived. The underlying FBL breakup mechanism occurs at a certain threshold value of the drive, $F_{c}$. We estimated $F_{c}$ by equating the FBL width, δ, with the net displacement undergone by a driven tracer while being advected across a convection roll, $F/4\Omega_{L}$; hence [22]:

(5) $$\begin{array}{c} F_{c}/U_{0}=4\sqrt{D_{0}/D_{L}}. \end{array}$$

More numerical evidence of the critical nature of the dynamical transition taking place at $F∼F_{c}$ will be provided in an upcoming full-length paper [33].

## 3. Diffusion Anisotropy

**Figure 2:**
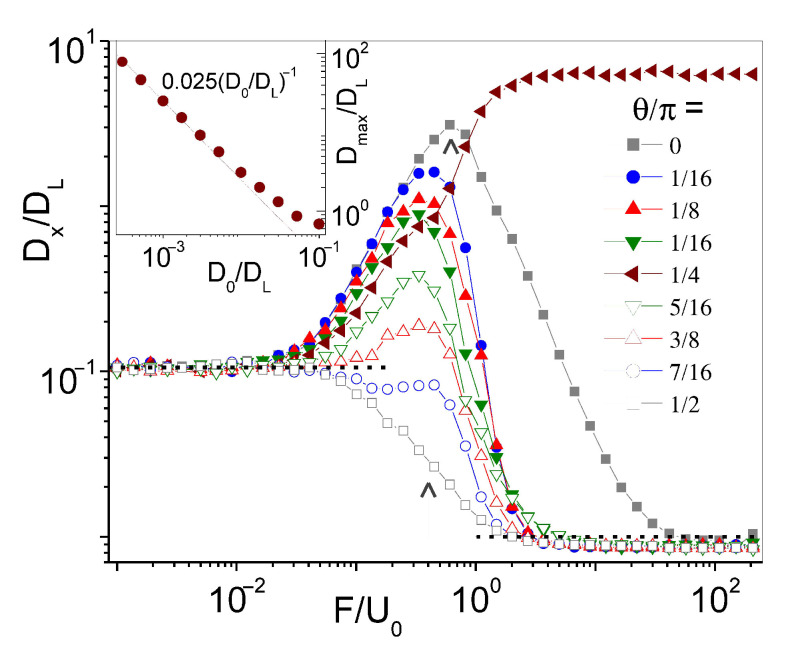
Longitudinal diffusion of a driven particle in a square convection array of Equation (1): $D_{x}/D_{L}$ vs. $F/U_{0}$ for $D_{0}=0.01$ and different drive orientations (see legend). Dashed lines on the left- and right-hand sides are respectively the predicted AED, $\kappa \sqrt{D_{L}D_{0}}$, and free diffusion constant, $D_{0}$. The vertical arrows denote our estimates for the FBL breakup threshold, $F_{c}=0.4$, Equation (5), and the position of the excess diffusion peak, $F_{m}=0.64$, Equation (6). Inset: power-law fit of $D_{\max}$ vs. $D_{0}$ for θ=0; see Equation (8). The flow parameters are $U_{0}=1$, L=2π, with $D_{L}=1$.

The main results of this work are illustrated in Figure 2, where several $D_{x}\left(F,\theta \right)$ curves are plotted versus the force intensity, *F*, for increasing values of the angle θ in the interval [0,π/2]. A few properties of the longitudinal diffusion constant for Pe≫1 are noteworthy:

(i) The limits F→0 and F→∞ of all curves coincide respectively with the AED, $D_{x}=\kappa \sqrt{D_{L}D_{0}}$, and the free diffusion constant, $D_{x}=D_{0}$. While the former limit was anticipated and discussed in Section 2, the horizontal asymptotes for $F\gg U_{0}$ set in when advection grows negligible with respect to the external drive, so that the driven particle diffuses effectively advection-free [34].

(ii) Most $D_{x}\left(F,\theta \right)$ curves exhibit huge excess diffusion peaks for values of *F* ranging between $F_{c}$, Equation (5), and:

(6) $$\begin{array}{c} F_{m}/U_{0}=2/\pi . \end{array}$$

$F_{m}$ denotes the position of the diffusion peak for θ=0 and can be estimated by equating the drag time across a ψ(x,y) unit cell, $\tau_{F}=L/F$, with the advection drag time around a roll, i.e., a circle of radius L/4, $\tau_{\psi }=\left(\pi L/2\right)/U_{0}$ [22].

We also observe that the height of the excess diffusion peaks in Figure 2, $D_{\max}$, is inverse proportional to the noise strength, $D_{0}$ (see the inset), and diminishes with increasing θ from zero to π/2.

(iii) The profile of the $D_{x}$ curves described in (ii) holds for all force orientations with three remarkable exceptions: (1) θ=0, where the diffusion peak is the most pronounced and so broad that $D_{x}$ overshoots $D_{0}$ also for $F\gg U_{0}$. (2) θ=π/2, where $D_{x}$ bridges the AED and free-diffusion constants smoothly, without going through a maximum. Its step-like profile is centered at $F_{c}$. We recall that $D_{x}\left(\pi /2\right)=D_{y}\left(0\right)$ is the transverse diffusion constant corresponding to the longitudinal diffusion peak for θ=0. (3) θ=π/4, where longitudinal and transverse diffusion constants coincide and grow monotonically with *F* until they level off to form a plateau, which can be higher than the diffusion peak at θ=0.

With these exceptions, the diffusion peaks illustrated in Figure 2 can be regarded as a manifestation of the so-called excess diffusion mechanism [35,36,37]. Let us consider diffusion in the *x* direction. The applied force, $\vec{F}$, has a twofold action. Its transverse component, $F_{y}=F\sin\theta$, helps break up the advection FBL, thus forcing the particle to diffuse along easy flow paths represented by the horizontal ψ(x,y) separatrices, y=±nπ, with *n* an integer. Its longitudinal component, $F_{x}=F\cos\theta$, pulls the particle along the above horizontal paths, contrasted by advection. Longitudinal diffusion is then modeled by the effective Langevin equation $\dot{x}=U_{0}\langle \cos\left(2\pi y/L\right)\rangle \sin\left(2\pi y/L\right)+F_{x}+\xi_{x}\left(t\right)$; see Equation (2). This approximate equation holds good under the assumption that after FBL breakup, the *x* and *y* coordinates can be regarded as decoupled. At low noise, Pe≫1, the particle thus moves in the longitudinal direction subject to the advection washboard potential of amplitude $D_{L}\langle \cos\left(2\pi y/L\right)\rangle$. Depinning occurs for $F≳U_{0}\langle \cos\left(2\pi y/L\right)\rangle$ and is marked by a conspicuous enhancement of the relevant diffusion constant, $D_{x}$. Along the easy flow paths, |〈cos(2πy/L)〉|=1. The magnitude of this effect, termed excess diffusion, in a washboard potential is inversely proportional to the noise strength, $D_{0}$ [37]. In the present case, this argument is necessarily restricted to drive intensities with $F_{c}<F<U_{0}$, in agreement with our simulation results.

The case of an advected particle driven diagonally across the convection rolls is rather unique and is treated in Section 4. The broad diffusion peak of a particle dragged parallel to the main axes of the square convection array is the focus of Section 5.

## 4. Diagonal Diffusion

**Figure 3:**
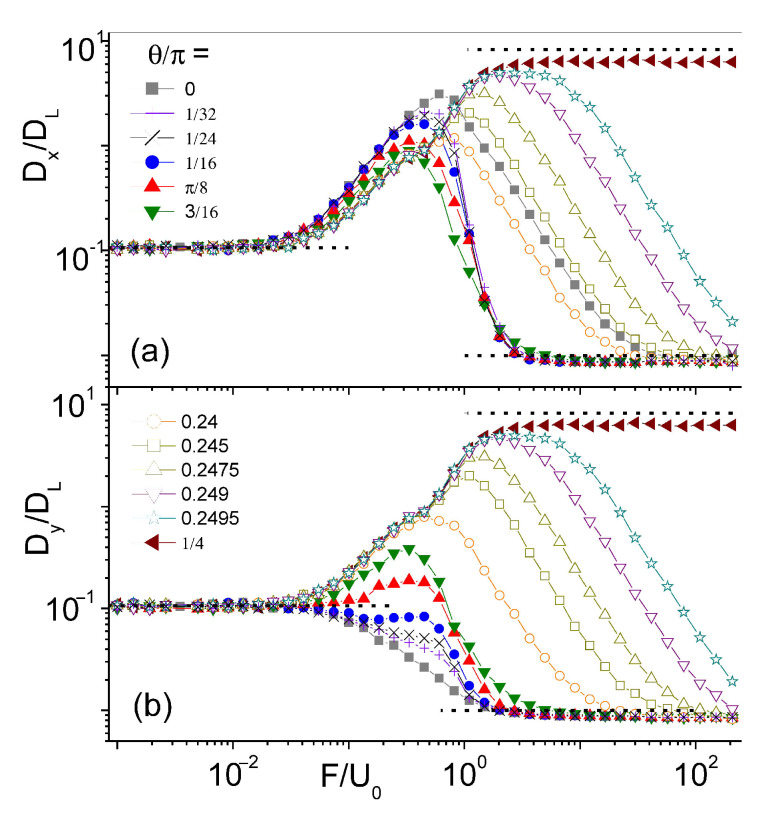
Diagonal diffusion constants of a driven particle in a square convection array of Equation (1): (**a**) $D_{x}/D_{L}$ and (**b**) $D_{y}/D_{L}$ vs. $F/U_{0}$ for θ close to zero and π/4 (legend split between the two panels) and $D_{0}=0.01$. Dashed lines on the left- and right-hand sides and all remaining simulation parameters are as in Figure 2. The topmost dashed line is the predicted asymptote of Equation (7).

Driven diffusion in the diagonal directions across the convection rolls, θ=±π/4, is characterized by a unique *F*-dependence of the diffusion constant. As shown in Figure 3, the curves $D_{x}\left(F,\pi /4\right)=D_{y}\left(F,\pi /4\right)$ grow asymptotically independently of *F*, up to a very high plateau. This points to a specific diffusion mechanism, which only holds in a narrow θ interval centered around θ=±π/4. In particular, we checked that this effect does not occur along any other axes of the square convection array, that is for tanθ=m/n, with *m* and *n* coprime integers different from one. What makes the diagonal directions so special? Let us consider a couple of parallel straight paths, $y=\alpha x\pm y_{0}$, with α=tanθ and $0\leq y_{0}\leq L/4$ (Figure 1a), and take the average, ${\bar{u}}_{\theta }$, of the advection velocity along them, $u_{\theta }=u_{x}\left(x,\alpha x\pm y_{0}\right)\cos\theta +u_{y}\left(x,\alpha x\pm y_{0}\right)\sin\theta$, with $u_{x}$ and $u_{y}$ defined in Equation (2). A simple integration with respect to *x* yields $\bar{u}/U_{0}=\pm \sin\left(2\pi y_{0}/L\right)/\sqrt{2}$, for α=±1, and $\bar{u}/U_{0}=0$ otherwise.

This result suggests that the particle is advected in the opposite direction along any two parallel paths with offset $\pm y_{0}$. Accordingly, upon neglecting advection for $F\gg U_{0}$ and low noise, $D_{0}\ll D_{L}$, diffusion in the diagonal direction is mostly due to path switching. Indeed, propelled by thermal noise, the particle switches paths by diffusing the distance $y_{0}/\sqrt{2}$ between them in a time of the order of [34] $\tau \left(y_{0}\right)=y_{0}^{2}/4D_{0}$. This results in a contribution $D_{x}\left(y_{0}\right)=\bar{u}{\left(y_{0}\right)}^{2}\tau \left(y_{0}\right)/4$ to the longitudinal diffusion constant. Finally, averaging $D_{x}\left(y_{0}\right)$ with respect to $y_{0}$ in the interval [0,L/4] yields:

(7) $$\begin{array}{c} \frac{D_{x}}{D_{L}}=K_{d}\left(\frac{D_{L}}{D_{0}}\right), \end{array}$$

with $K_{d}=\pi^{2}/32\left(1/6+1/\pi^{2}\right)≃0.083$. Of course, our average applies to any pair of adjacent diagonal paths vertically spaced by multiples of L/2.

**Figure 4:**
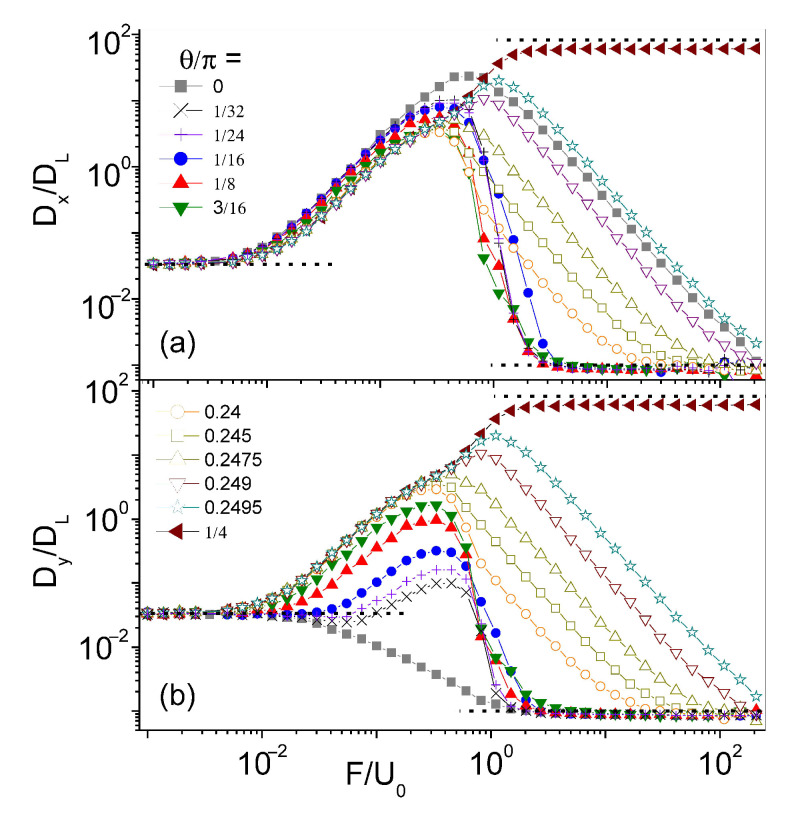
Same as Figure 3, except $D_{0}=0.001$.

Our estimate, Equation (7), for the asymptote of $D_{x}\left(F,\pi /4\right)$ is in close agreement with the simulation data of Figure 3. In Figure 4, we plot the curves of $D_{x}\left(F,\pi /4\right)$ obtained for the same parameter values, except the noise strength was lowered by one order of magnitude. As expected, $D_{x}\left(F,\pi /4\right)$ approaches a plateau ten times higher as an effect of the path switching mechanism advocated above.

Such a mechanism should not be mistaken for the excess diffusion mechanism recalled in Section 3. Indeed, the latter requires that the transverse component of $\vec{F}$ is large enough to pull the particle out of the top (bottom) longitudinal FBL branches, which happens for $|\theta |>\theta_{c}$ with $\theta_{c}=F_{c}/U_{0}$ and $F_{c}$ given in Equation (5) [33]. On the contrary, the path switching mechanism dominates in a small θ interval around θ=±π/4, $\Delta \theta <F_{c}/U_{0}$. Our estimate for Δθ is consistent with the data of Figure 3 and Figure 4, where Δθ appears to shrink with increasing $D_{0}$.

## 5. Longitudinal and Transverse Diffusion

**Figure 5:**
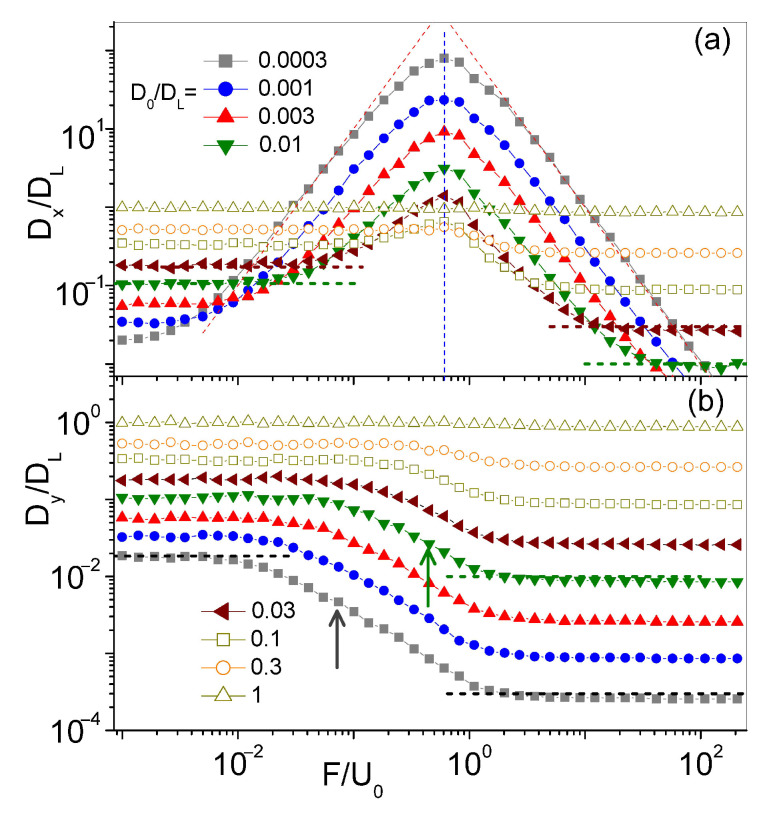
Longitudinal and transverse diffusion constants of a particle driven in the convection array of Equation (1) at θ=0: (**a**) $D_{x}/D_{L}$ and (**b**) $D_{y}/D_{L}$ vs. $F/U_{0}$ for different $D_{0}$ (legend split between the two panels). Dashed oblique curves are drawn by fitting Equation (8) with $K_{0}=0.025\pm 0.005$ and $F^{*}=0.60\pm 0.01$ (denoted by a vertical dashed line). Dashed horizontal lines represent the limits F→0 and F→∞, like in Figure 2, for two different values of $D_{0}$; the relevant estimates of $F_{c}$, Equation (5), are marked in (**b**) by vertical arrows of the same color. Other simulation parameters are: L=2π, $U_{0}=1$, with $D_{L}=1$.

Advection impacts longitudinal (θ=0) and transverse diffusion (θ=±π/2), in a distinct manner, as illustrated in Panels (a) and (b) of Figure 5. Simulation data for $D_{x}\left(F,0\right)$ and $D_{y}\left(F,0\right)$ at high Péclet numbers are plotted versus $F/U_{0}$, for different values of $D_{0}$. A few remarkable properties are apparent by inspection [22]:

(i) Both diffusion curves approach the respective limits $\kappa \sqrt{D_{0}D_{L}}$ for F→0 and $D_{0}$ for F→∞, as already noticed in Figure 2. The curves of the transverse diffusion constant, $D_{y}\left(F,0\right)$, exhibit a smooth, step-like profile centered at $F=F_{c}$, whereas the longitudinal diffusion peaks are much broader than the excess diffusion peaks observed for θ≠0,π/4 in Figure 2.

(ii) As anticipated in Section 3, the curves $D_{x}\left(F,0\right)$ peak around the same value of the force intensity, $F_{m}$, for a wide interval of the noise strength, $D_{0}$. Indeed, based on the definition of $F_{m}$, Equation (6), for $F=F_{m}$, advection and external drag would nullify each other along one horizontal FBL branch, while doubling their action along the opposite one. Under this condition, the longitudinal diffusion constant is expected to attain a maximum [38]. Moreover, at low noise, the peak height, $D_{\max}$, is inverse proportional to $D_{0}^{-1}$, as reported in the inset of Figure 2.

(iii) The profiles of the $D_{x}\left(F,0\right)$ peaks are symmetric: Their left (right) sides rise (decay) proportional to $F^{2}$ ($F^{-2}$). A working fitting formula for the longitudinal diffusion peaks of Figure 5 is:

(8) $$\begin{array}{c} \frac{D_{x}}{D_{L}}=K_{0}\left(\frac{D_{L}}{D_{0}}\right){\left[\frac{2F/F^{*}}{1+{\left(F/F^{*}\right)}^{2}}\right]}^{2}, \end{array}$$

where $K_{0}$ and $F^{*}$ are fitting parameters. Our best fits in Figure 5 are obtained for $F^{*}=0.60\pm 0.01$, which is close to the expected value of Equation (6). The small discrepancy can be attributed to the action of thermal noise, which, at high Péclet numbers, differs significantly in the weak and strong drive regimes.

The fitting formula in Equation (8) can be interpreted by means of a heuristic argument. The advection flows along the top and bottom array edge are spatially modulated with period *L* and opposite in phase. Moreover, when trying to apply the edge switching approach leading to Equation (7), we notice that the average of the advection drag, $\bar{u}\left(\pm y_{0}\right)$, taken along the edges of a longitudinal channel of width $2y_{0}$, with $0\leq y_{0}\leq L/4$, vanishes (see, e.g., Figure 1b). The ensuing edge switching process can then be regarded as an alternating renewal process [39]. Accordingly, to calculate $D_{x}$ for θ=0, we need to replace [22]:

$$\frac{u_{\theta }}{U_{0}}\to \frac{{\left(F+u_{x}\right)}^{2}-{\left(F-u_{x}\right)}^{2}}{{\left(F+u_{x}\right)}^{2}+{\left(F-u_{x}\right)}^{2}}.$$

Finally, the spatial averages over a channel unit cell yield a result qualitatively consistent with the fitting formula of Equation (8).

## 6. Conclusions

The response of an overdamped Brownian particle driven-advected in a 2D square convection array shows large deviations from the predictions of the linear response theory. While the mobility is independent of the drive orientation, its diffusion turns out to be strongly anisotropic. Depending on the drive orientation, the diffusion constants along the main array’s axes exhibit distinct peaks for advection and external drags of comparable magnitude.

The anisotropic diffusion process investigated in this work should not be mistaken with the anisotropic dynamics of a driven Brownian particle suspended in a fluid at rest in a 2D lattice of obstacles of a given shape [13,14,15] or in other constrained geometries [40,41]. In the present case, the response of a tracer particle is strongly affected by the stationary advection currents. Measuring its diffusion constants along special directions, parallel and diagonal to the array axes, allows a direct characterization of the convection pattern at hand. As mentioned in the Introduction, the generalizations of the fluctuation-dissipation theorem have been proposed [3], which allow, at least in principle, a more refined analytic treatment of this problem. Interesting, in this respect, also is the multiscale scheme developed in [42].

As a natural extension of the approach presented in this paper, we plan to investigate the anisotropic response of active Brownian particles next, either biological or synthetic, in convection arrays [27,43,44]. We are confident that a better understanding of the diffusion of self-propelling particles in patterned convection flows is likely to play an important role in controlling the driven transport of active matter in microfluidic devices.

## Data Availability

Data are available upon reasonable request from all the authors.

## Abbreviations

The following abbreviations are used in this manuscript:

AED	advection enhanced diffusion
FBL	flow boundary layer
